# Impact of insulin resistance on mild cognitive impairment in type 2 diabetes mellitus patients with non-alcoholic fatty liver disease

**DOI:** 10.1186/s13098-023-01211-w

**Published:** 2023-11-10

**Authors:** Hui Zhang, Huzaifa Fareeduddin Mohammed Farooqui, Wenwen Zhu, Tong Niu, Zhen Zhang, Haoqiang Zhang

**Affiliations:** 1 Henan Key Laboratory of Rare Diseases, Endocrinology and Metabolism Center, The First Affiliated Hospital, and College of Clinical Medicine of Henan University of Science and Technology,, Luoyang, China; 2 Department of Endocrinology, Affiliated Zhongda Hospital of Southeast University, Nanjing, China; 3 Department of Endocrinology, Centre for Leading Medicine and Advanced Technologies of IHM, The First Affiliated Hospital of USTC, Division of Life Sciences and Medicine, University of Science and Technology of China, Hefei, China

**Keywords:** Type 2 Diabetes Mellitus, Non-alcoholic fatty Liver Disease, Mild cognitive impairment

## Abstract

**Aims:**

Insulin resistance (IR) is a pivotal factor in the pathogenesis of type 2 diabetes mellitus (T2DM) and non-alcoholic fatty liver disease (NAFLD). Nevertheless, the impact of IR on cognitive dysfunction in T2DM patients with NAFLD remains inadequately understood. We aim to investigate the effect of IR on mild cognitive impairment (MCI) in T2DM individuals with NAFLD.

**Materials and methods:**

143 T2DM individuals were categorized into Non-MCI and MCI groups, as well as Non-NAFLD and NAFLD groups. Clinical parameters and cognitive preference test outcomes were compared. Correlation and regression analyses were executed to explore the interconnections between IR and cognitive details across all T2DM patients, as well as within the subgroup of individuals with NAFLD.

**Results:**

In comparison to the Non-MCI group, the MCI group displayed elevated HOMA-IR levels. Similarly, the NAFLD group exhibited higher HOMA-IR levels compared to the Non-NAFLD group. Additionally, a higher prevalence of MCI was observed in the NAFLD group as opposed to the Non-NAFLD group. Notably, HOMA-IR levels were correlated with Verbal Fluency Test (VFT) and Trail Making Test-B (TMTB) scores, both related to executive functions. Elevated HOMA-IR emerged as a risk factor for MCI in the all patients. Intriguingly, increased HOMA-IR not only correlated with TMTB scores but also demonstrated an influence on TMTA scores, reflecting information processing speed function in patients with NAFLD.

**Conclusion:**

IR emerges as a contributory factor to cognitive dysfunction in T2DM patients. Furthermore, it appears to underlie impaired executive function and information processing speed function in T2DM individuals with NAFLD.

## Introduction

With the progressively escalating incidence of type 2 diabetes mellitus (T2DM) [1, 2], its complications, encompassing cognitive impairments, have garnered escalating attention amongst researchers [3]. Diabetic cognitive dysfunction encompasses mild cognitive impairment (MCI) and dementia [4, 5]. Presently, the global dementia patient population nears 50 million, and with the exacerbation of aging trends, this number is projected to surpass 130 million by the year 2025 [6]. Consequently, preemptive intervention to avert the onset of dementia assumes paramount significance. The stage of MCI represents a pivotal juncture amenable to intervention [7]. Elucidating its pathogenic mechanisms assumes critical significance, as this elucidation stands to facilitate early-stage assessment of the disease condition, screening for potential intervention targets, and prevention of dementia occurrence.

Our prior research has previously demonstrated a correlation between MCI associated T2DM and insulin resistance (IR) [8]. Notably, IR constitutes a pivotal mechanistic underpinning in the pathogenesis and progression of T2DM [9]. Furthermore, individuals afflicted by T2DM concomitant with IR exhibit an augmented susceptibility towards non-alcoholic fatty liver disease (NAFLD) [10, 11]. Beyond its potential evolution into non-alcoholic steatohepatitis (NASH), cirrhosis, hepatocellular carcinoma [12], and even into extrahepatic tumor, like the bladder cancer [13], NAFLD has also been implicated in the impairment of cognitive function [14, 15].

NAFLD is not only implicated in the development of T2DM [16], but is also closely associated with IR [17, 18]. Beyond IR, additional pathological mechanisms of NAFLD may contribute to the onset of cognitive dysfunction. These encompass alterations in gut microbiota composition, oxidative stress, inflammatory responses, lipid metabolism, and notably, cholesterol metabolism [19].

Indeed, meta-analytical investigations have demonstrated that patients with NAFLD exhibit a risk for cognitive impairment exceeding 1.44-fold in comparison to healthy controls [20]. Radiological examinations have revealed that individuals with NAFLD manifest reduced cerebral volume, heightened arterial sclerosis, and compromised cerebral blood flow when contrasted with the general population [21–23]. Liver histopathology stands as one of the most precise criteria for diagnosing and assessing the severity of fatty liver disease. A study employing liver biopsy as a diagnostic measure has delineated a correlation between the extent of hepatocellular ballooning and the global cognitive function as assessed by the Mini-mental state examination (MMSE) score [24]. Furthermore, supplementary findings from functional magnetic resonance imaging (fMRI) have illuminated a linkage between NAFLD and cognitive function, whereby the severity of NAFLD is intricately associated with hippocampal impairments [25].

Despite numerous investigations having demonstrated the involvement of IR in NAFLD, T2DM, and cognitive impairments, the precise role of IR in patients afflicted by T2DM concomitant with NAFLD remains to be elucidated comprehensively. This is particularly imperative in the context of accounting for the adjustment of confounding factors such as age, gender, body mass index (BMI), glycemic and lipid metabolic status, and so on.

## Methods

### Experimental design

**Fig. 1:**
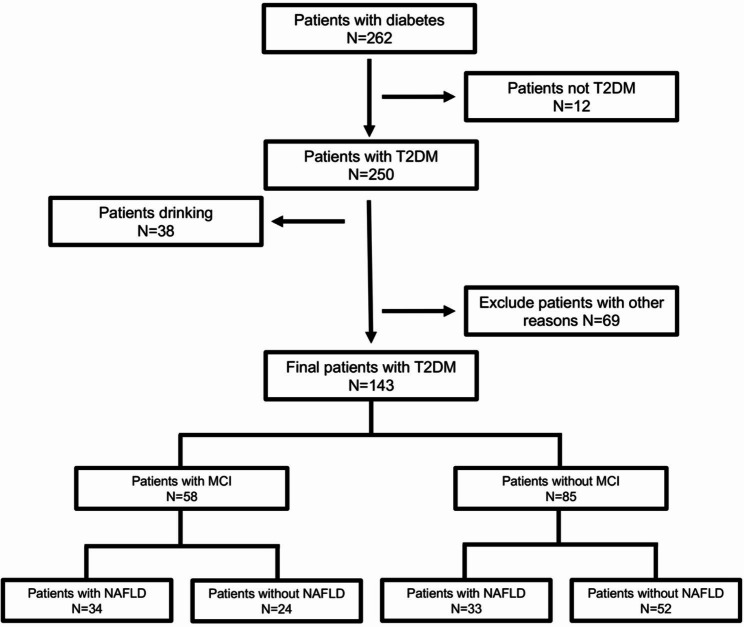
The workflow chart of this study **Notes**: other reasons including: (**a**) one or multiple other liver diseases including virus infection of hepatitis, diseases of biliary obstructive or autoimmune hepatitis; (**b**) any drug usage may influence the function of liver, like: tamoxifen, amiodarone, sodium valproate, methotrexate, and glucocorticoids etc.; (**c**) recent diagnosed acute complications of diabetes; (**d**) severe low plasma glucose; (**e**) acute vascular disease of heart and brain; **e**) drug abuse; (**f**) diagnosed disease of thyroid (with thyroid dysfunction or abnormal autoimmune antibodies); (**g**) severe infection, major surgery, (**h**) visual or hearing dysfunction (cannot finish neuropsychological tests); (**i**) dementia (severe cognitive decline out of the range of MCI); **k**) other diseases may affect (or potentially influence) cognition; cognitive function testing and inflammation, like anemia, cancer, and autoimmune disease (e.g., Crohn’s disease, rheumatoid arthritis, systemic lupus erythematosus, and so on)

In the current study, a total of 143 participants was recruited from the Endocrinology Department, The First Affiliated Hospital of USTC. These individuals were rigorously assessed for eligibility based on the established criteria outlined by the World Health Organization for the diagnosis of T2DM [26]. The workflow chart was described in Fig. 1. Among these participants, 58 were identified as having concomitant MCI in accordance with the diagnostic criteria proposed by the MCI Working Group of the European Consortium on Alzheimer’s Disease [27]. The remaining 85 individuals demonstrated an unimpaired cognitive function and were designated as Non-MCI subjects. The diagnostic criteria employed for NAFLD were consistent with the guidelines stipulated for the Asia-Pacific region [28] and described briefly as the presence of at least two out of three abnormal findings on abdominal ultrasonography: a liver that appears diffusely hyperechoic with echogenicity surpassing that of the kidney or spleen, vascular blurring, and deep attenuation of ultrasound signal. The likelihood of NAFLD is significantly high when other potential causes of liver disease, especially notable alcohol consumption (exceeding 140 g per week in men, 70 g per week in women), and medication use, have been thoroughly ruled out.

### Ethics

All participants were duly informed about the present study and subsequently provided their informed consent by affixing their signatures. This cross-sectional investigation obtained ethical approval from the Ethics Committee of The First Affiliated Hospital of USTC (Approval No.:2023-RE-292).

### Inclusion and exclusion criteria

The inclusion criteria for this study were specifically defined as follows: participant had to be inpatient with a duration of diabetes exceeding 3 years and aged 45 years or older. Conversely, the exclusion criteria were delineated, building upon our prior investigation into diabetic cognitive impairment [29]. In particular, additional exclusion standards were established for this study, which focused on NAFLD: (a) alcohol consumption exceeding 140 g per week in men, 70 g per week in women; (b) having one or multiple other liver diseases including virus infection of hepatitis, diseases of biliary obstructive or autoimmune hepatitis; (c) any drug usage may influence the function of liver, like: tamoxifen, amiodarone, sodium valproate, methotrexate, and glucocorticoids etc. Subsequently, patients were categorized into two groups based on the absence or presence of MCI: the non-MCI group and the MCI group. Similarly, patients were divided into the non-NAFLD and NAFLD groups based on the absence or presence of NAFLD.

### Clinical data collection

The information of age, gender, high, weight, systolic and diastolic pressures as well as the duration of diabetes mellitus (DM) and hypertension of all patients was collected. BMI was calculated by Weight (kg)/height (m)^2^. The data of fasting plasma glucose (FPG), serum c-peptide, HbA1c, Triglyceride (TG), total cholesterol (TC), high-density lipoprotein cholesterol (HDL-C), and low-density lipoprotein cholesterol (LDL-C), alanine aminotransferase (ALT) and aspartate aminotransferase (AST), was also isolated from the medical history. HOMA-IR was calculated by 1.5 + FBG (mmol/L) × FCP (pmol/L)/2800 [30]. Both systolic and diastolic pressures were measured at the second day when these patients were hospitalized.

### Neuropsychological tests

All neuropsychological tests were described as our previous study [31]. The global cognitive function was assessed by Montreal cognitive assessment (MoCA). Additionally, 1 score was added as the history of education was < 12 years. The executive function of individuals was measured by digit span test (DST), VFT and TMTB. Visual space function was observed by clock drawing test (CDT). TMTA test was performed to evaluate the function of information processing speed. Auditory verbal learning test-immediate recall (AVLT-IR) and auditory verbal learning test-delayed recall (AVLT-DR) were conducted to exam the instantaneous memory function and delayed memory function, respectively. The function of scene memory was detected by logical memory test (LMT).

### Statistical methods

Data was analyzed using SPSS 26.0 (IBM, USA). The data of systolic pressure and diastolic pressure, cholesterol levels including TC, HDL-C, LDL-C, as well as cognitive performance scores including TMTA and AVLT-I scores are normally distributed variables. These data were described as mean ± standard deviation. Their differences were compared by Student’s t test. The data of Age, BMI, Duration of DM and hypertension, FPG, HbA1c, HOMA-IR and TG, ALT and AST, levels as well as cognitive performance scores including MoCA, DST, VFT, CDT, TMTB, AVLT-L and LMT scores were asymmetrically distributed variables. These data were described as median (interquartile range). Their differences were compared by nonparametric Mann–Whitney U. the data of gender and the diagnosis of NAFLD or MCI were binary variables. Their differences were compared by chi-squared test. Pearson and partial correlation analyses, as well as binary logistic analyses, and multiple linear regression were performed to investigate the association between HOMA-IR and MCI (and the cognitive performance details) in all patients and those with NAFLD. P < 0.05 was considered as statistically significant.

## Results

First and foremost, a comparative analysis of clinical parameters was conducted between patients with and without MCI. It was observed that there were no statistically significant differences in age and gender between the two groups (all P > 0.05). Similarly, there were no statistically significant distinctions found in BMI, duration of DM and hypertension, as well as systolic and diastolic blood pressure levels, when comparing patients in the MCI group with those in the Non-MCI group (all P > 0.05). To assess glucose metabolism, data pertaining to FPG and HbA1c levels were collected, and the HOMA-IR was calculated. A comparison of these parameters between the two groups revealed that while FPG levels were higher in diabetic patients with MCI compared to those without MCI, this difference did not reach statistical significance (P > 0.05). Interestingly, the levels of HbA1c and HOMA-IR were significantly elevated in patients with MCI as compared to those in the Non-MCI group (P = 0.031). Additionally, to assess lipid metabolism, levels of TG, TC, LDL-C, and HDL-C were measured. However, no statistically significant differences were found in the levels of these lipid constituents between the two groups (all P > 0.05). As a pivotal aspect of this study, the prevalence of NAFLD in these two groups was also compared. It was observed that there was a higher frequency of NAFLD among patients in the MCI group (34 out of 58) as compared to the Non-MCI group (33 out of 85) (P = 0.020) as the diagnostic tools used described above in methods section for NAFLD [28] (Table 1).

**Table 1:** Comparation of clinical parameters and neurophysiological test results between Non-MCI group and MCI group

	Non-MCI group (n = 85)	MCI group (n = 58)	P
Age (years)	60.00 (55.00-73.50)	59.00 (54.00-66.35)	0.818^b^
Female (n, %)	40, 40.06	22, 37.93	0.279^c^
BMI (kg/m^2^)	24.41 (22.58–25.71)	24.77 (22.38–27.12)	0.706^b^
Duration of DM (years)	10.00 (6.50–18.00)	10.00 (7.00-15.50)	0.208^b^
Duration of HBP (years)	0.00 (0.00–10.00)	0.00 (0.00-5.25)	0.875^b^
Systolic pressure (mmHg)	131.01 ± 16.96	135.07 ± 20.42	0.141^a^
Diastolic pressure (mmHg)	78.49 ± 9.95	81.90 ± 11.54	0.062^a^
FPG (mmol/l)	6.80 (5.51–8.34)	8.18 (6.21–10.05)	0.109^b^
HbA1c (%)	7.90 (6.90–9.15)	8.80 (6.88–9.73)	0.031^b*^
HOMA-IR	2.38 (2.16–2.90)	2.74 (2.22–3.21)	0.009^b^
TG (mmol/l)	1.41 (0.92–1.97)	1.70 (0.97–2.50)	0.207^b^
TC (mmol/l)	4.32 ± 0.98	4.45 ± 1.37	0.554^a^
HDL-C (mmol/l)	1.09 ± 0.31	1.04 ± 0.30	0.395^a^
LDL-C (mmol/l)	2.52 ± 0.78	2.57 ± 1.00	0.759^a^
NAFLD (n, %)	33, 38.82	34, 58.62	0.020^c*^
MoCA	29.00 (28.00–30.00)	24.00 (24.00–25.00)	< 0.001^b*^
DST	12.00 (10.00–13.00)	10.00 (9.00–12.00)	0.032 ^b*^
VFT	17.00 (14.00-21.50)	13.00 (9.00–17.00)	< 0.001^b*^
CDT	4.00 (3.00–4.00)	3.00 (2.00–3.00)	< 0.001^b*^
TMTA	60.14 ± 16.06	67.50 ± 20.38	0.017^a*^
TMTB	125.00 (100.00-182.00)	176.50 (128.50-219.75)	0.003 ^b*^
AVLT-IR	18.72 ± 4.80	16.29 ± 4.49	0.003^a*^
AVLT-DR	6.00 (5.00–8.00)	5.00 (4.00-6.25)	0.028^b*^
LMT	11.00 (7.00–14.00)	8.00 (6.00–10.00)	0.004^b*^

Notes: ^a^ Student’s t test was employed for normally distributed variables; ^b^ The Mann-Whitney U test was employed for asymmetrically distributed variables; ^c^ The Chi-square test was employed for categorical variables. ^*^ P < 0.05; ^**^ P < 0.01. Abbreviations: MCI, mild cognitive impairment; BMI, body mass index; DM, diabetes mellitus; HBP, high blood pressure; FPG, fasting plasma glucose; HbA1c, glycosylated hemoglobin; TG, triglyceride; TC, total cholesterol; HDL-C, high-density lipoprotein cholesterol; LDL-C, low-density lipoprotein cholesterol; NAFLD, non-alcoholic fatty liver disease; MoCA, Montreal cognitive assessment; DST, digit span test; VFT, verbal fluency test; CDT, clock drawing test; TMTA, trail making test-A; TMTB, trail making test-B; AVLT-IR, auditory verbal learning test-immediate recall; AVLT-DR, auditory verbal learning test-delayed recall; LMT, logical memory test

As depicted in Table 1, individuals within the MCI cohort manifested diminished scores across a range of cognitive assessments, namely the MoCA, DST, VFT, CDT, AVLT-IR, AVLT-DR, and LMT, in comparison to their counterparts in the Non-MCI group. However, when compared with diabetic patients devoid of MCI, subjects afflicted with MCI exhibited significantly reduced scores on the TMTA and TMTB (all P < 0.05). These findings not only validate the presence of global cognitive impairment in MCI patients but also elucidate the specific nuances of cognitive impairment in this diabetic population.

Due to variations in HOMA-IR levels observed among patients with and without MCI, an examination of the correlation between HOMA-IR and cognitive function test scores was undertaken. Initially, Pearson correlation analysis was conducted, revealing significant correlations between HOMA-IR levels and scores on the VFT and TMTB in the entire patient cohort (P = 0.007 and 0.002, respectively) (described as model 1). Furthermore, partial correlation analyses were performed, accounting for covariates (although it should be noted that there were no statistically significant differences observed, potentially due to limitations arising from non-matched basic information inherent in a cross-sectional study design). After adjusting for variables such as age, gender, duration of DM, and hypertension, HOMA-IR levels remained significantly correlated with VFT and TMTB scores (P = 0.009 and 0.003, respectively) (described as model 2). Moreover, even after incorporating HbA1c levels as adjusting factors (which are elevated in patients with MCI), the association between HOMA-IR levels and VFT, as well as HOMA-IR and TMTB scores, persisted (P = 0.012 and 0.004, respectively) (described as model 3) (see Table 2).

**Table 2:** Association between HOMA-IR and cognitive functions in patients with T2DM

	Model 1		Model 2		Model 3	
	R	P	R	P	R	P
MoCA	-0.116	0.168	-0.131	0.124	-0.120	0.161
DST	-0.111	0.187	-0.096	0.262	-0.093	0.276
VFT	-0.226	0.007^**^	-0.221	0.009^**^	-0.214	0.012^*^
CDT	-0.037	0.664	-0.013	0.876	-0.024	0.780
TMTA	0.086	0.306	0.078	0.364	0.056	0.511
TMTB	0.261	0.002^**^	0.253	0.003^**^	0.241	0.004^**^
AVLT-IR	-0.111	0.185	-0.122	0.152	-0.115	0.178
AVLT-DR	-0.139	0.098	-0.154	0.070	-0.150	0.079
LMT	-0.028	0.741	-0.041	0.630	-0.041	0.631

Notes: Model 1 showed the Pearson association between HOMA-IR and cognitive function in patients with T2DM; Model 2 showed the partial correlation between HOMA-IR and cognitive function adjusting for age, gender, duration of DM and hypertension; Model 3 showed the partial correlation between HOMA-IR and cognitive function adjusting for age, gender, duration of DM and hypertension as well as HbA1c. ^*^ P < 0.05; ^**^ P < 0.01. Abbreviations: T2DM, type 2 diabetes mellitus; MoCA, Montreal cognitive assessment; DST, digit span test; VFT, verbal fluency test; CDT, clock drawing test; TMTA, trail making test-A; TMTB, trail making test-B; AVLT-IR, auditory verbal learning test-immediate recall; AVLT-DR, auditory verbal learning test-delayed recall; LMT, logical memory test; DM, diabetes mellitus; HbA1c, glycosylated hemoglobin

In order to comprehensively ascertain the risk factors associated with MCI in individuals diagnosed with T2DM, binary logistic regression analyses were conducted. It was observed that HOMA-IR emerged as a significant risk factor for MCI in T2DM patients (P = 0.039; OR = 1.563) (model 1). Further analysis found that HOMA-IR is still the risk factor of MCI after adjusting for age, gender, duration of DM and hypertension without (model 2) or with (model 3) HbA1c also added in the adjusting factors (P = 0.019, OR = 1.702; P = 0.037; OR = 1.623, respectively) (refer to Table 3).

**Table 3:** Assessment of risk factors for MCI in patients with T2DM by binary logistic analysis

	P	OR	95% CL of OR	
Model 1				
HOMA-IR	0.039^*^	1.563	1.023	2.388
Model 2				
Age	0.191	0.972	0.931	1.014
Gender	0.356	1.400	0.686	2.857
Duration of DM	0.781	1.008	0.954	1.065
Duration of HBP	0.300	0.973	0.925	1.024
HOMA-IR	0.019^*^	1.702	1.090	2.657
Model 3				
Age	0.318	0.978	0.936	1.022
Gender	0.356	1.401	0.685	2.866
Duration of DM	0.931	1.003	0.947	1.061
Duration of HBP	0.352	0.976	0.927	1.027
HbA1c	0.304	1.114	0.907	1.369
HOMA-IR	0.037^*^	1.623	1.029	2.560

Notes: Model 1 showed that HOMA-IR is the risk factor for MCI in patients with T2DM; Model 2 showed that HOMA-IR is the risk factor for MCI in patients with T2DM adjusting for age, gender, duration of DM and hypertension; Model 3 showed that HOMA-IR is the risk factor for MCI in patients with T2DM adjusting for age, gender, duration of DM and hypertension as well as HbA1c. ^*^ P < 0.05; ^**^ P < 0.01. Abbreviations: MCI, mild cognitive impairment; T2DM, type 2 diabetes mellitus; DM, diabetes mellitus; HBP, high blood pressures; HbA1c, glycosylated hemoglobin

In Table 4, a comparative analysis was conducted to assess the cognitive performance characteristics among patients with and without NAFLD. Similar to the findings presented in Table 1, it was observed that the frequency of MCI was notably higher in individuals with NAFLD (34 out of 76) as compared to participants without NAFLD (24 out of 76) (P = 0.020). However, there were no significant differences observed in the MoCA scores between the NAFLD and non-NAFLD groups. In order to gain a more comprehensive understanding of the cognitive profiles in patients with and without NAFLD, additional assessments were performed, encompassing the following cognitive measures: DST, VFT, CDT, AVLT-IR, AVLT-DR, TMTA, TMTB, and LMT. Notably, the VFT scores indicated a lower level of executive function in patients with NAFLD compared to those without NAFLD (P = 0.009). Conversely, the scores for DST, CDT, AVLT-IR, AVLT-DR, TMTA, TMTB, and LMT did not demonstrate any statistically significant differences between the two groups (all P > 0.05).

**Table 4:** Comparation of neurophysiological test results between Non-NAFLD group and NAFLD group

	Non-NAFLD group (n = 76)	NAFLD group (n = 67)	P
MCI (n, %)	24, 31.58	34, 50.75	0.020^c*^
MoCA	28.00 (25.00–29.00)	26.00 (25.00–29.00)	0.985^b^
DST	11.00 (10.00–13.00)	11.00 (10.00–13.00)	0.481^b^
VFT	17.00 (13.00-21.75)	14.00 (12.00–17.00)	0.009^b**^
CDT	3.00 (2.00–4.00)	3.00 (3.00–4.00)	0.534^b^
TMTA	61.74 ± 18.42	64.70 ± 18.03	0.334^a^
TMTB	136.00 (100.75-180.75)	162.00 (116.00-224.00)	0.278^b^
AVLT-IR	18.32 ± 4.88	17.07 ± 4.68	0.124^a^
AVLT-DR	6.00 (5.00–7.00)	5.00 (4.00–7.00)	0.625^b^
LMT	9.00 (7.00–13.00)	9.00 (6.00–11.00)	0.402^b^

Notes: ^*^ P < 0.05; ^**^ P < 0.01; Abbreviations: NAFLD, non-alcoholic fatty liver disease; MCI, mild cognitive impairment; MoCA, Montreal cognitive assessment; DST, digit span test; VFT, verbal fluency test; CDT, clock drawing test; TMTA, trail making test-A; TMTB, trail making test-B; AVLT-IR, auditory verbal learning test-immediate recall; AVLT-DR, auditory verbal learning test-delayed recall; LMT, logical memory test

**Fig. 2:**
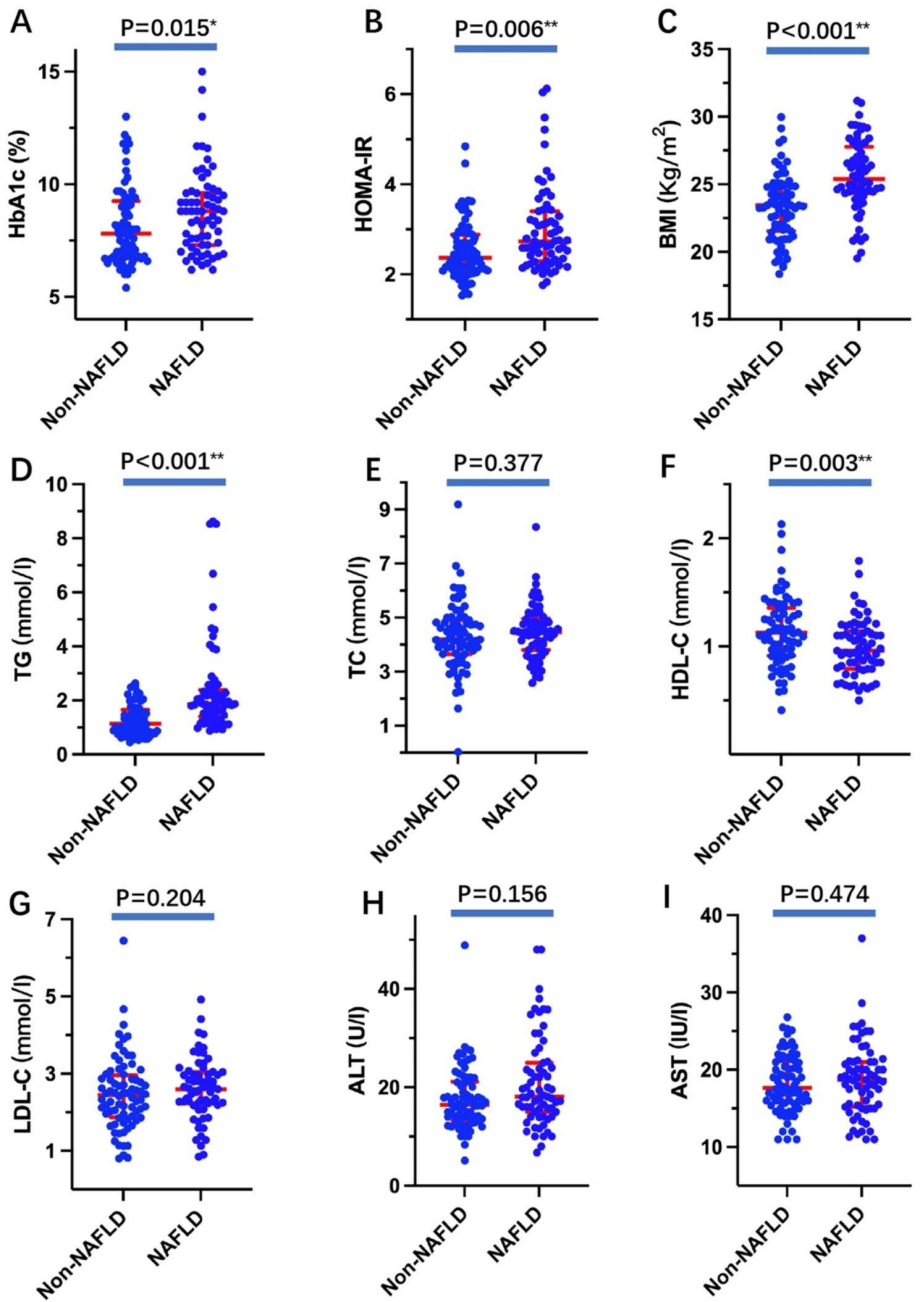
Comparation of clinical parameters between Non-NAFLD group and NAFLD group **Notes**: ^*^ P < 0.05; ^**^ P < 0.01; (**A**) Comparation of HbA1c levels between Non-NAFLD group and NAFLD group; (**B**) Comparation of HOMA-IR between Non-NAFLD group and NAFLD group; (**C**) Comparation of BMI between Non-NAFLD group and NAFLD group; (**D**) Comparation of TG levels between Non-NAFLD group and NAFLD group; (**E**) Comparation of TC levels between Non-NAFLD group and NAFLD group; (**F**) Comparation of HDL-C levels between Non-NAFLD group and NAFLD group; (**G**) Comparation of LDL-C levels between Non-NAFLD group and NAFLD group; (**H**) Comparation of ALT levels between Non-NAFLD group and NAFLD group; (**I**) Comparation of AST levels between Non-NAFLD group and NAFLD group. Abbreviations: NAFLD, non-alcoholic fatty liver disease; HbA1c, glycosylated hemoglobin; BMI, body mass index; TG, triglyceride; TC, total cholesterol; HDL-C, high-density lipoprotein cholesterol; LDL-C, low-density lipoprotein cholesterol

As delineated in the aforementioned context, a notable disparity in the levels of HbA1c and HOMA-IR was observed, with significantly higher values discerned among patients afflicted with MCI in comparison to those devoid of this condition. These differential levels were also subjected to scrutiny within the subset of patients afflicted by NAFLD and those free from its manifestation, the results of which are succinctly presented in Fig. 2A and B. Remarkably, a pronounced elevation in these metrics was additionally ascertained in patients diagnosed with diabetes mellitus and co-occurring NAFLD when juxtaposed with their counterparts lacking NAFLD (all P < 0.05). Furthermore, as the investigation encompassed NAFLD, a comparative analysis was conducted on various metabolic parameters, including BMI, TG, TC, LDL-C, HDL-C, ALT, and AST. Of notable interest, it was discerned that elevated BMI and TG levels were concomitant with a concurrent reduction in HDL-C levels (all P < 0.05). However, no discernible distinctions were noted in other indexes (all P > 0.05) (Fig. 2C, D, E, D and G H, and 2I).

In a manner analogous to the comprehensive analysis undertaken in all patients diagnosed with T2DM, an examination of the associations between HOMA-IR and neuropsychological test outcomes was performed in a subset of diabetic individuals presenting with NAFLD. Utilizing Pearson’s correlation coefficient, the analysis revealed a statistically significant positive correlation between HOMA-IR and performance scores on TMTA (P = 0.005) and TMTB (P = 0.003) in this particular patient cohort (model 1). Notably, when adjusting for covariates including age, gender, duration of DM, and the presence of hypertension without (model 2) or with (model 3) HbA1c, HOMA-IR levels exhibited associations not only with TMTA (P = 0.04) and TMTB scores (P = 0.07), but also with AVLT-IR scores (P = 0.046) as demonstrated through partial correlation analysis (P = 0.039). However, when BMI, TG, and HDL-C were introduced as additional adjusting factors, the previously observed relationship between HOMA-IR and AVLT-IR scores became non-significant (P = 0.122) (model 4), as elucidated in Table 5.

**Table 5:** Association between HOMA-IR and cognitive functions in T2DM patients with NAFLD.

	Model 1			Model 2		Model 3		Model 4	
		R	P	R	P	R	P	R	P
MoCA		-0.156	0.206	-0.183	0.152	-0.165	0.199	-0.004	0.975
DST		-0.138	0.267	-0.177	0.165	-0.174	0.175	-0.095	0.437
VFT		-0.183	0.139	-0.189	0.137	-0.176	0.172	-0.108	0.414
CDT		-0.167	0.176	0.154	0.228	-0.193	0.133	-0.084	0.528
TMTA		0.334	0.005^**^	-0.355	0.004^**^	0.342	0.007^**^	0.279	0.032^*^
TMTB		0.354	0.003^**^	0.371	0.003^**^	0.363	0.004^**^	0.348	0.007^**^
AVLT-IR		-0.192	0.120	-0.225	0.076	-0.231	0.071	-0.182	0.167
AVLT-DR		-0.236	0.055	-0.253	0.046^*^	-0.263	0.039^*^	-0.204	0.122
LMT		-0.126	0.310	-0.088	0.495	-0.115	0.372	-0.054	0.684

Notes: Model 1 showed the Pearson association between HOMA-IR and cognitive function in pa-tients with T2DM; Model 2 showed the partial correlation between HOMA-IR and cognitive function adjusting for age, gender, duration of DM and hypertension; Model 3 showed the partial correlation between HOMA-IR and cognitive function adjusting for age, gender, duration of DM and hypertension as well as HbA1c; Model 4 showed the partial correlation between HOMA-IR and cognitive function adjusting for age, gender, duration of DM and hypertension, HbA1c, BMI, TG, and HDL-C. ^*^ P < 0.05; ^**^ P < 0.01. Abbreviations: T2DM, type 2 diabetes mellitus; NAFLD, non-alcoholic fatty liver disease; MoCA, Montreal cognitive assessment; DST, digit span test; VFT, verbal fluency test; CDT, clock drawing test; TMTA, trail making test-A; TMTB, trail making test-B; AVLT-IR, au-ditory verbal learning test-immediate recall; AVLT-DR, auditory verbal learning test-delayed recall; LMT, logical memory test; DM, diabetes mellitus; HbA1c, glycosylated hemoglobin; BMI, body mass index; TG, triglyceride; HDL-C, high-density lipoprotein cholesterol

To further explore the impact of HOMA-IR on cognitive function, a detailed investigation was undertaken employing multiple linear regression analyses. The objective was to elucidate the effect of HOMA-IR on TMTA and TMTB scores within a cohort of diabetic patients afflicted with NAFLD. Intriguingly, the analysis revealed that HOMA-IR not only exerted a statistically significant influence on TMTA scores (P = 0.032) but also on TMTB scores (P = 0.007). Importantly, this effect was observed to be independent of other potentially confounding factors such as age, gender, duration of DM, hypertension, HbA1c, BMI, TG, and HDL-C levels in patients with T2DM who also had NAFLD, as presented in Table 6.

**Table 6:** Analysis for factors influence the TMTA and TMTB scores of T2DM patients with NAFLD.

	TMTA				TMTB			
	P	β	95% CI for β		P	β	95% CI for β	
Age	0.293	0.375	-0.317	1.302	0.756	-0.373	-2.762	2.016
Gender	0.678	1.989	7.547	11.526	0.628	-8.216	-41.989	25.558
Duration of DM	0.463	-0.262	-0.972	0.488	0.337	-1.217	-3.732	1.298
Duration of HBP	0.399	-0.273	-0.917	0.371	0.682	-0.469	-2.750	1.811
HbA1c	0.911	0.143	-2.408	2.695	0.768	-1.340	-10.375	7.696
BMI	0.486	0.639	-1.187	2.464	0.308	3.320	-3.145	9.786
TG	0.956	-0.078	-2.927	2.771	0.193	-6.636	-16.726	3.455
HDL	0.158	-12.485	-29.959	4.989	0.894	-4.127	-66.011	57.757
HOMA-IR	0.032^*^	5.911	0.532	11.450	0.007^**^	27.077	7.742	46.411

Notes: ^*^ P < 0.05; ^**^ P < 0.01; Abbreviations: TMTA, trail making test-A; TMTB, trail making test-B; T2DM, type 2 diabetes mellitus; NAFLD, non-alcoholic fatty liver disease; DM, diabetes mellitus; HBP, high blood pressure; HbA1c, glycosylated hemoglobin; BMI, body mass index; TG, triglyceride; HDL-C, high-density lipoprotein cholesterol

## Discussion

Many studies have posited a potential relationship between IR and cognitive function, both in populations without diabetes [8, 32, 33], with pre-diabetes [34] and with comorbid diabetes [35–37]. T2DM patients notably exhibit IR as a prominent characteristic. Thus, IR emerges as a focal point of investigation within this study. Indeed, our research initially reveals that T2DM patients with coexisting cognitive impairment exhibit a more pronounced degree of IR when compared to a control group. Further correlational analyses within the overall study population demonstrate a significant association between the HOMA-IR levels, indicative of IR, and the patients’ performance in cognitive function assessments, specifically the VFT and the TMTB scores. These findings collectively suggest that IR may be implicated in the cognitive decline observed in patients with T2DM, particularly in the context of impaired executive function. Importantly, these findings align with previous research results. A large sample size, 11 years follow-up study identifies IR as a potential predictive factor for cognitive impairment [38]. Furthermore, research has revealed disturbances in glucose metabolism within specific brain regions in both cognitively normal middle-aged adults and individuals with prediabetes, mirroring patterns observed in severe cognitive impairment in Alzheimer’s disease patients [39]. Additionally, more in-depth investigations have shown reduced insulin receptor density in the brains of individuals with cognitive decline, along with impairment in downstream insulin signaling pathways. Moreover, insulin signaling pathways are known to exert neuroprotective effects and contribute to synaptic plasticity, thus potentially ameliorating cognitive function [40].

In addition to T2DM, IR stands out as a prominent feature of NAFLD [41]. An investigation conducted in the United States, featuring a large-scale sample, revealed that individuals afflicted with NAFLD exhibited impaired cognitive function [42]. Our research findings parallel those of this aforementioned study. Specifically, our investigation underscores a heightened probability of MCI in individuals with coexisting T2DM and NAFLD. Indeed, laboratory research has elucidated that high-calorie diets inducing NAFLD model exhibit memory decline, associated with hippocampal dysfunction [43]. However, it is worth noting that these animal models not only manifest NAFLD but also exhibit metabolic aberrations in tissues such as adipose and muscle, potentially encompassing damage to the central nervous system, including hippocampal tissue. Additional functional magnetic resonance imaging results indicate a correlation between the severity of NAFLD and cognitive impairment, which is linked to structural and functional abnormalities within the hippocampus [25].

As previously elucidated, extensive research has been undertaken to explore the impact of IR on cognitive dysfunction in individuals with diabetes, and there is also evidence suggesting that NAFLD may influence cognitive function. While a recent research posits that IR and NAFLD are associated with cognitive performance in pre-diabetic individuals and newly diagnosed type 2 diabetes patients who are obese (BMI > 30 kg/m^2^) [44], there remains a dearth of systematic studies examining cognitive function changes in T2DM patients with co-occurring NAFLD. Our investigation not only reveals a higher probability of MCI in patients with concomitant NAFLD, but also demonstrates a decline in executive function among this subgroup. In addition to comparing changes in executive function between T2DM patients with and without NAFLD, we have also analyzed the correlation between IR and cognitive function in different populations. In all patients with T2DM, there exists a correlation between IR and executive function. However, in T2DM patients with co-occurring NAFLD, IR is not only associated with executive function but also with information processing speed function. This correlation persists even after adjusting for factors such as age, gender, duration of DM and hypertension, HbA1c levels, BMI, TG, and HDL-C. Firstly, the observation that the relationship between IR and cognitive function varies across different populations suggests that the liver’s role in mediating the impact of IR on cognitive function is pivotal within the context of T2DM. Secondly, in T2DM patients with NAFLD, the influence of IR on cognitive function may be independent of factors such as glycemic control, BMI, and lipid control, and so on.

In this study, the first step substantiates prior assertions, confirming the impact of IR on cognitive dysfunction within the population of T2DM patients. Furthermore, we provide the inaugural evidence of an exploration into the relationship between IR and MCI within type 2 diabetes patients who also suffer from NAFLD. We delineate the similarities and disparities in this relationship between the broader T2DM population and those with concomitant NAFLD. These distinctions shed light on the potential role of NAFLD in the context of cognitive dysfunction associated with diabetes. Indeed, NAFLD may exert its influence on cognitive function through multiple pathways. Firstly, as a crucial metabolic organ, the liver plays a pivotal role in processes such as glucose metabolism, lipid metabolism, and notably, cholesterol metabolism. Previous research has posited associations between glucose metabolism [45–47], cholesterol metabolism [31, 48], and cognitive function. Secondly, the liver harbors a rich population of macrophages, and activated macrophages may impact cognitive function through the secretion of inflammatory cytokines. Prior studies have underscored the significant role of inflammatory responses in the development and progression of cognitive function impairments [49, 50]. Moreover, during hepatic metabolism, various oxidative stress factors may be generated, which could potentially participate in the chronic complications of diabetes [51], including cognitive dysfunction [52]. Furthermore, the liver may directly influence neurological changes via the liver-brain axis [19, 53, 54]. Lastly, hepatic metabolic processes, including active hepatocytes, are known to produce extracellular vesicles, and these vesicles may carry bioactive components that can traverse the blood-brain barrier, thus directly affecting cognitive function within the central nervous system [55].

Although our study has provided the first-ever insights into the impact of IR on cognitive function in T2DM patients with concurrent NAFLD, it is essential to acknowledge several limitations inherent to our research. Firstly, our study was a cross-sectional analysis, thus rendering our conclusions as merely indicative of correlations rather than establishing causation. The specific causal relationships require further validation through cohort studies. Additionally, through analysis of variance and subsequent pairwise comparisons of HOMA-IR levels among different groups (MCI + NAFLD group, MCI + non-NAFLD group, non-MCI + NAFLD group, and non-MCI + non-NAFLD group), a more comprehensive elucidation of the potential role of insulin resistance in cognitive dysfunction and NAFLD becomes feasible. Although we contemplated presenting the results in this manner, the constraints of our small-sample cross-sectional study precluded such an approach as it would yield limited sample sizes. Consequently, we adopted a two-step strategy to present our findings. In the initial step, we compared HOMA-IR levels between T2DM patients with and without concomitant cognitive dysfunction, aiming to discern the potential involvement of IR in diabetes-related cognitive impairment. Subsequently, in the second step, we conducted a similar analysis comparing HOMA-IR levels between T2DM patients with and without coexisting NAFLD, seeking insights into the plausible role of IR in NAFLD. We acknowledged the limitation inherent in this analytical approach due to the relatively inadequate sample size. Secondly, our study encountered some unwell-matched data that was rectified in subsequent analyses. It remains plausible that these discrepancies could have influenced the ultimate outcomes. In addition, gender may exert an influence on cognitive function [56], but our study did not discern any notable disparities in this regard. This pattern extends to the impact of gender on NAFLD, perhaps attributable to the heterogeneity of our study. While our investigation yields such findings, it is imperative to acknowledge that we do not refute the potential influence of gender on cognitive function, recognizing our study as one among many. As numerous meta-analyses culminate in profoundly meaningful conclusions by assimilating a plethora of studies, encompassing both positive and negative outcomes, we refrain from negating the role of gender based on our lack of observed distinctions. Admittedly, intriguing results may emerge from subgroup analyses, but due to limitations in sample size, we refrained from exhaustive subgroup analyses. In subsequent investigations, gender was appropriately adjusted for, mitigating potential confounding effects. Thirdly, visceral adiposity exhibits a close association with cognitive impairment in diabetes [57]. The inclusion of waist circumference or waist-hip ratio data would have conferred greater significance to our study. Regrettably, due to the retrospective cross-sectional nature of our research, pertinent data of such metrics is currently unavailable. Instead, our analysis relied solely on the computation of Body Mass Index (BMI) using patients’ height and weight. We acknowledge this as a limitation in our study. Forth, the impact of anti-diabetic medications on cognitive function in patients is substantiated. However, this detailed medication categorization was not explicitly presented in the manuscript due to several reasons. (1) although we collected information on the current usage categories of anti-diabetic medications for each patient, the specific dosage and duration of medication use were not systematically recorded. (2) despite accounting for the medication regimen of each patient, a notable proportion of the subjects were concurrently utilizing multiple anti-diabetic agents. Consequently, the association between cognitive and each medication is difficult to explore in this study. While further subgroup analyses could potentially address these concerns, the limited sample size posed a challenge. Some medications are only being used by a very small number of patients. We acknowledge this as a limitation in our study. Furthermore, to supplement this gap, we incorporated pertinent findings from a network meta-analysis conducted by our former colleagues, which meticulously delineated the cognitive repercussions associated with each medication category [58]. Fifth, in order to mitigate the potential impact of smoking on study outcomes, all individuals with a history of smoking were excluded from the study. Nevertheless, it is noteworthy that extant research has posited the conceivable impact of secondhand smoke on complications related to diabetes. Ideally, this study should have incorporated an assessment of exposure to secondhand smoke. Regrettably, the retrospective nature of the study precluded the availability of data on secondhand smoke exposure. Furthermore, physical activity, another critical variable, should ideally have been taken into account. However, our study faced a similar constraint in lacking comprehensive data on the physical activity levels of the patients. The absence of data pertaining to secondhand smoke exposure and physical activity has been duly acknowledged as limitations of the study. Inflammation plays a pivotal role in cognitive dysfunction associated with diabetic complications [29, 59, 60]. The measurement of C-response protein or other inflammation markers in the blood may be interesting. However, we lack the information of these factors for this is a retrospective study. We have to address that this is one of the limitations. Lastly, the mechanistic pathways through which IR affects cognitive function in the context of NAFLD remain unexplored and warrant further investigation through fundamental research endeavors.

## Conclusion

IR emerges as a significant contributing factor to cognitive dysfunction in individuals diagnosed with T2DM. Moreover, it appears to underlie the impairment of executive function and information processing speed in T2DM patients who also exhibit NAFLD. The management of IR, encompassing both systemic and hepatic IR, as well as the treatment of NAFLD, holds promise as potential therapeutic targets in the context of addressing cognitive dysfunction in patients with T2DM.

## Data Availability

All data in this manuscript have been submitted to The First Affiliated Hospital of USTC for records. The ID of recruited patients was also collected for further usage. All data are available on reasonable request from corresponding authors.
